# Tip Detection–Antegrade Dissection and Re-Entry With New Puncture Wire in CTO Intervention: Revolution Through 3D-Wiring

**DOI:** 10.1016/j.jacasi.2023.11.017

**Published:** 2024-02-27

**Authors:** Kota Tanaka, Atsunori Okamura, Ryouhei Yoshikawa, Etsuo Tsuchikane, Masato Ishikawa, Satoshi Suzuki, Hiroyuki Nagai, Akinori Sumiyoshi, Masatsugu Kawahira, Tomohiro Yamasaki, Hiroaki Matsuda, Mutsumi Iwamoto, Satoshi Watanabe, Keita Yamasaki, Nobuaki Tanaka, Yasushi Koyama, Yoshitaka Iwanaga, Heitaro Watanabe

**Affiliations:** a Cardiovascular Center, Sakurabashi Watanabe Hospital, Osaka, Japan; b Sanda City Hospital, Sanda, Japan; c Department of Cardiovascular Medicine, Toyohashi Heart Center, Toyohashi, Japan; d Department of Cardiology, Fujita Health University, Aichi, Japan; e Kindai University Nara Hospital, Nara, Japan; f Department of Cardiovascular Medicine, Nagoya Heart Center, Nagoya, Japan

**Keywords:** antegrade dissection re-entry, chronic total occlusion, coronary intervention, IVUS-based 3D wiring, tip detection method

## Abstract

**Background:**

The authors devised the tip detection (TD) method and developed AnteOwl WR intravascular ultrasound to standardize intravascular ultrasound–based 3-dimensional wiring for intraplaque tracking in chronic total occlusion (CTO)–percutaneous coronary intervention (PCI). The TD method also allowed antegrade dissection and re-entry (ADR). Combining TD-ADR with Conquest Pro 12 Sharpened Tip (CP12ST) wire, a new ADR wire with the strongest penetration force developed to date, enabled re-entry anywhere except calcification sites.

**Objectives:**

This study investigated the efficacy and feasibility of TD-ADR by comparison of procedural outcomes with Stingray-ADR in CTO-PCI.

**Methods:**

Twenty-seven consecutive CTO cases treated by TD-ADR with CP12ST wire between August 2021 and April 2023 and 27 consecutive CTO cases treated by Stingray-ADR with Conquest 8-20 (CP20) wire between March 2018 and July 2021 were retrospectively enrolled as the TD-ADR by CP12ST wire group and Stingray-ADR by CP20 wire group, respectively, from 4 facilities that could share technical information on these procedures.

**Results:**

The success rate of the ADR procedure was significantly improved (27 of 27 cases [100%] vs 18 of 27 cases [67%], respectively; *P* = 0.002) and total procedural time was significantly reduced (median procedural time: 145.0 [Q1-Q3: 118.0-240.0] minutes vs 185.0 [Q1-Q3: 159.5-248.0] minutes, respectively; *P* = 0.028) in the TD-ADR by CP12ST wire group compared to the Stingray-ADR by CP20 wire group. There were few in-hospital major adverse cardiac and cerebrovascular events or no complications in either group.

**Conclusions:**

TD-ADR by CP12ST wire can standardize highly accurate ADR in CTO-PCI.

In chronic total occlusion (CTO)–percutaneous coronary intervention (PCI), if guidewires cannot be passed through the CTO lesion in angiography-based antegrade wire escalation, it is recommended to move on to the retrograde approach^1^ or antegrade dissection and re-entry (ADR) using the Stingray system (current device-based ADR, Boston Scientific).^2^ These 2 strategies are effective, but have some problems. The retrograde approach uses a donor artery, which is a complex procedure and has a high risk of complications.^3^ Current device-based ADR has been widely used, but it does not have a high level of accuracy because this procedure uses angiographic observation.^2^ There is a lack of standardized accurate anterograde wiring techniques that should be performed before applying these 2 strategies.

To standardize the accurate anterograde wiring technique in CTO-PCI, over the last 13 years we have developed various methodologies and devices based on the 3-dimensional (3D) wiring method (Figure 1). In 2012, we developed the first CTO-specific intravascular ultrasound (IVUS) system, Navifocus WR-IVUS (Terumo Corp),^4^^,^^5^ and we realized the importance of 3D wiring and established angiography-based 3D wiring (Figure 1A).^6^^,^^7^ Subsequently, to standardize IVUS-based 3D wiring for intraplaque tracking, we devised a tip detection (TD) method and developed AnteOwl WR–intravascular ultrasound (AO-IVUS, Terumo Corp),^8^, ^9^, ^10^, ^11^ which is an upgraded version of Navifocus WR-IVUS with an added pullback transducer system.

**Figure 1:**
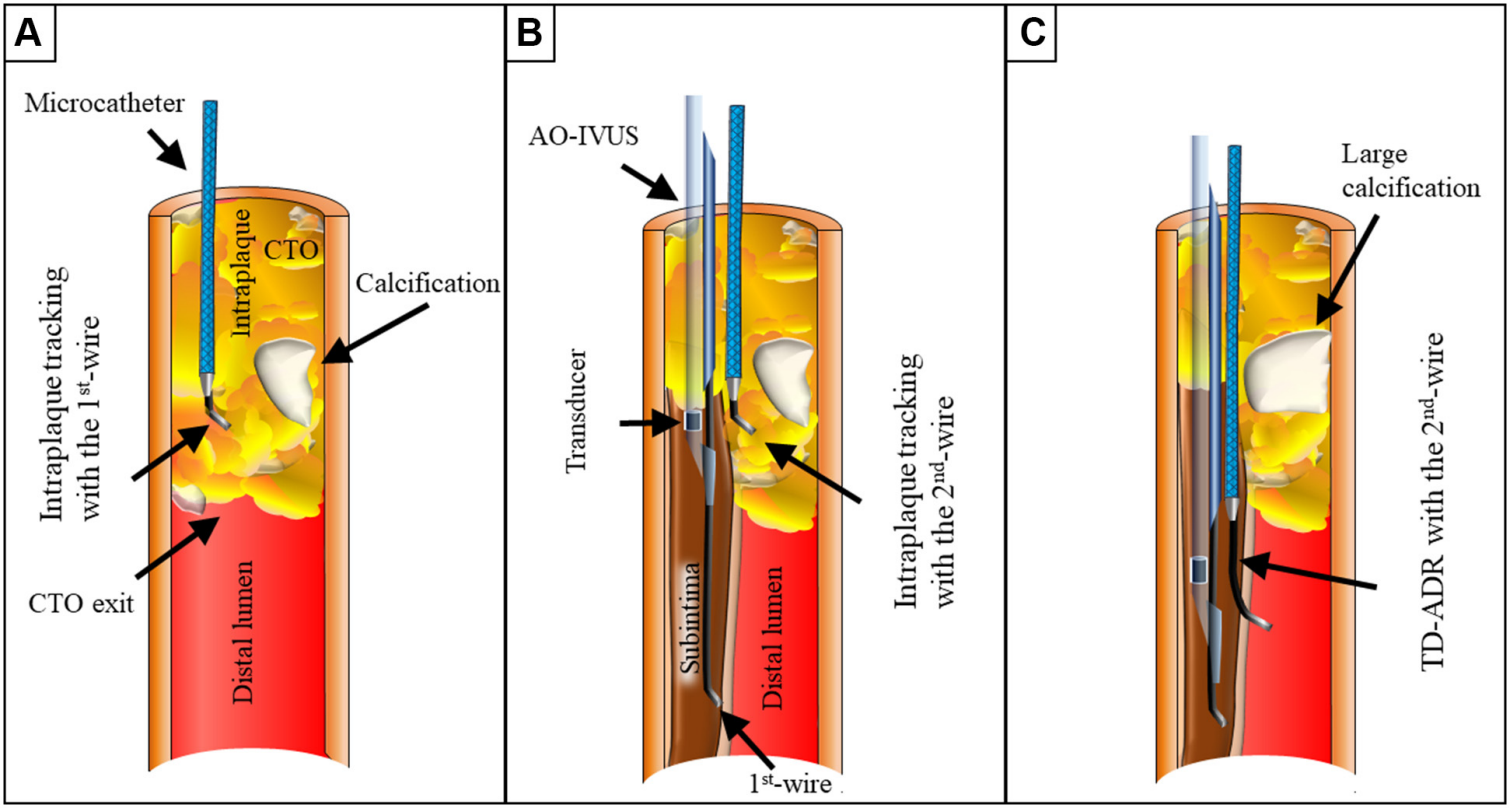
Angiography- and IVUS-Based 3D Wiring (A) Angiography-based 3-dimensional (3D) wiring, (B) intravascular ultrasound (IVUS)-based 3D wiring using the tip detection (TD)–intraplaque tracking, and (C) IVUS-based 3D wiring using the TD-antegrade dissection re-entry (ADR). AO-IVUS = AnteOwl WR intravascular ultrasound; CTO = chronic total occlusion.

Until recently, IVUS-guided wiring had been thought to be “intraplaque tracking” through the intraplaque route but not “re-entry” from the subintimal space to the true lumen. Therefore, we attempted to pass through the intraplaque route using the TD method (Figure 1B). However, in November 2021, we found that the TD method allows ADR because the wall between the subintima and the true lumen can be punctured at the intended site in an exactly vertical direction under IVUS observation.^12^ We named this method “tip detection–ADR” (TD-ADR) (Figure 1C).^13^ Compared with current device-based ADR, TD-ADR enables puncture at a more proximal site and allows pinpoint puncture at the intended site in the vertical direction because the target and the guidewire tip are clearly visible under IVUS observation (Central Illustration). In addition, in 2021, Conquest Pro 12 Sharpened Tip (CP12ST) wire, a new ADR wire with the strongest penetration force developed to date (Tsuchikane and Asahi Intecc Co, Ltd), became available.^14^ Combining TD-ADR with CP12ST wire enables re-entry anywhere at the distal lumen beyond the CTO lesion or even at the CTO body,^15^ except at sites of calcification (Central Illustration B).

**Central Illustration:**
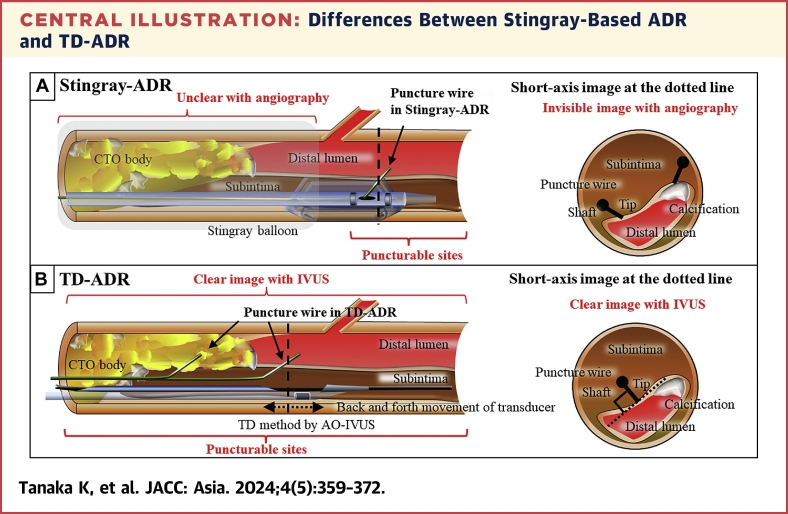
Differences Between Stingray-Based ADR and TD-ADR Illustrations of long-axis and short-axis procedures for (A) the current device-based antegrade dissection re-entry (ADR) including the Stingray-ADR and (B) AnteOwl WR intravascular ultrasound (AO-IVUS)–based ADR using the tip detection (TD) method (TD-ADR), respectively. The red words state the pros and cons of these procedures. CTO = chronic total occlusion.

In the present study, we compared the procedural outcomes of current device-based ADR by the conventional wire and TD-ADR by CP12ST wire in CTO-PCI.

## Methods

### Study population

We conducted a retrospective multicenter study of cases from Sakurabashi Watanabe Hospital (Osaka, Japan) and 3 other facilities—Sanda City Hospital (Sanda, Japan), Kindai University Nara Hospital (Nara, Japan), and Fujita Health University (Aichi, Japan)—that could share technical information on Stingray-based ADR and TD-ADR procedures.

We started TD-ADR by non-CP12ST wire in CTO-PCI in November 2020 and treated 4 cases by July 2021. As CP12ST wire was approved in August 2021, we performed TD-ADR by CP12ST wire in all TD-ADR cases. From August 2021 to April 2023, a total of 27 consecutive TD-ADR by CP12ST wire cases of 187 consecutive cases (the retrograde approach, 24 cases) undergoing CTO-PCI were retrospectively enrolled as the TD-ADR by CP12ST wire group.

In current device-based ADR, the stick was performed using the Stingray system with Conquest 8-20 (CP20) wire (Asahi Intecc Co, Ltd). The Stingray system was not performed after August 2021 because TD-ADR by CP12ST wire was shown to be highly reliable. Therefore, from March 2018 to July 2021, a total of 27 consecutive Stingray-based ADR cases of 317 consecutive cases (the retrograde approach, 48 cases) undergoing CTO-PCI were retrospectively enrolled as the Stingray-ADR by CP20 wire group.

The breakdown of the number of Stingray-ADR by CP20 wire cases was 21 in Sakurabashi Watanabe Hospital, 2 in Sanda City Hospital, 2 in Kindai University Nara Hospital, and 2 in Fujita Health University. The breakdown of the number of TD-ADR by CP12ST wire cases was 19 in Sakurabashi Watanabe Hospital, 3 in Sanda City Hospital, 4 in Kindai University Nara Hospital, and 1 in Fujita Health University. The study was approved by the Central Medical Ethics Committee of Sakurabashi Watanabe Hospital, Osaka, Japan (approval #23-02).

### Interventional procedures until ADR in CTO-PCI

A 7- or 8-F guide catheter was selected for the antegrade approach at the discretion of the operator. In antegrade wire escalation, the tip of the CTO stiff wires was 1 mm with a curve at an angle of 45°. A Corsair microcatheter (Asahi Intecc Co, Ltd) was mainly used because of sufficient backup support for the CTO guidewires. When the antegrade guidewire was advanced to be around the CTO exit but could not be passed through the CTO lesion due to advancement of the guidewire into the subintimal space, or intraplaque area other than the distal lumen, we changed to Stingray-ADR, IVUS-guided wiring, or the retrograde approach, while following the CTO-PCI algorithms.^16^^,^^17^

### Interventional procedures of current device-based ADR in CTO-PCI

In current device-based ADR, we followed the Japanese ADR technique advocated by Habara et al.^18^ Briefly, the stick-and-swap technique was attempted in all cases using the Stingray system with CP20 wire for stick and XT-R (Asahi Intecc Co, Ltd) for swap under angiographic guidance (Central Illustration A). After March 2018, when the Stingray balloon was delivered and inflated at the target position, aspiration was performed for 4 minutes using the guidewire lumen of the Stingray balloon to reduce the hematoma.

### Interventional procedures of TD-ADR in CTO-PCI

**Figure 2:**
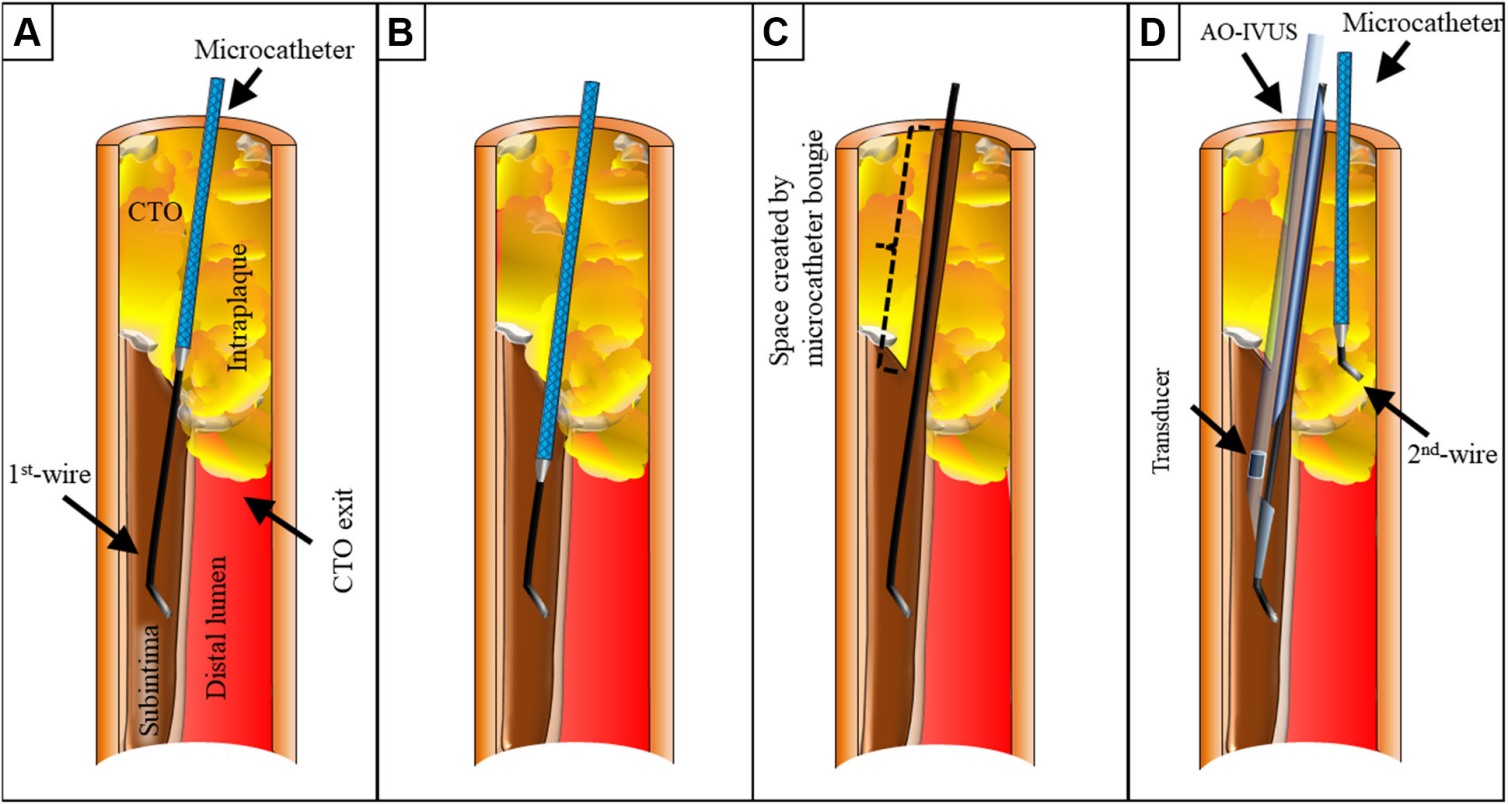
Procedural Flow Up to IVUS-Guided Wiring (A to D) Procedural flow from antegrade guidewire escalation to the starting point of IVUS-guided wiring. Abbreviations as in Figure 1.

In TD-ADR, when the first antegrade guidewire was advanced to be around the CTO exit but could not be passed through the CTO lesion due to advancement of the guidewire into the subintimal space, or intraplaque area other than the distal lumen, (Figure 2A), the Corsair was advanced through the guidewire around the CTO exit to create a space for the IVUS catheter (Figures 2B and 2C). If the guidewire was a tapered wire, it was changed to a 0.014-inch moderately stiff CTO wire to obtain good support for advancing the IVUS catheter. Using a double-chamber catheter, the second guidewire was then advanced and the microcatheter was advanced through this second wire, and the IVUS catheter was advanced through the first guidewire (Figure 2D). In addition, when the IVUS catheter could not be advanced after the Corsair’s bougie, balloon dilatation was performed with a small-diameter balloon.

**Figure 3:**
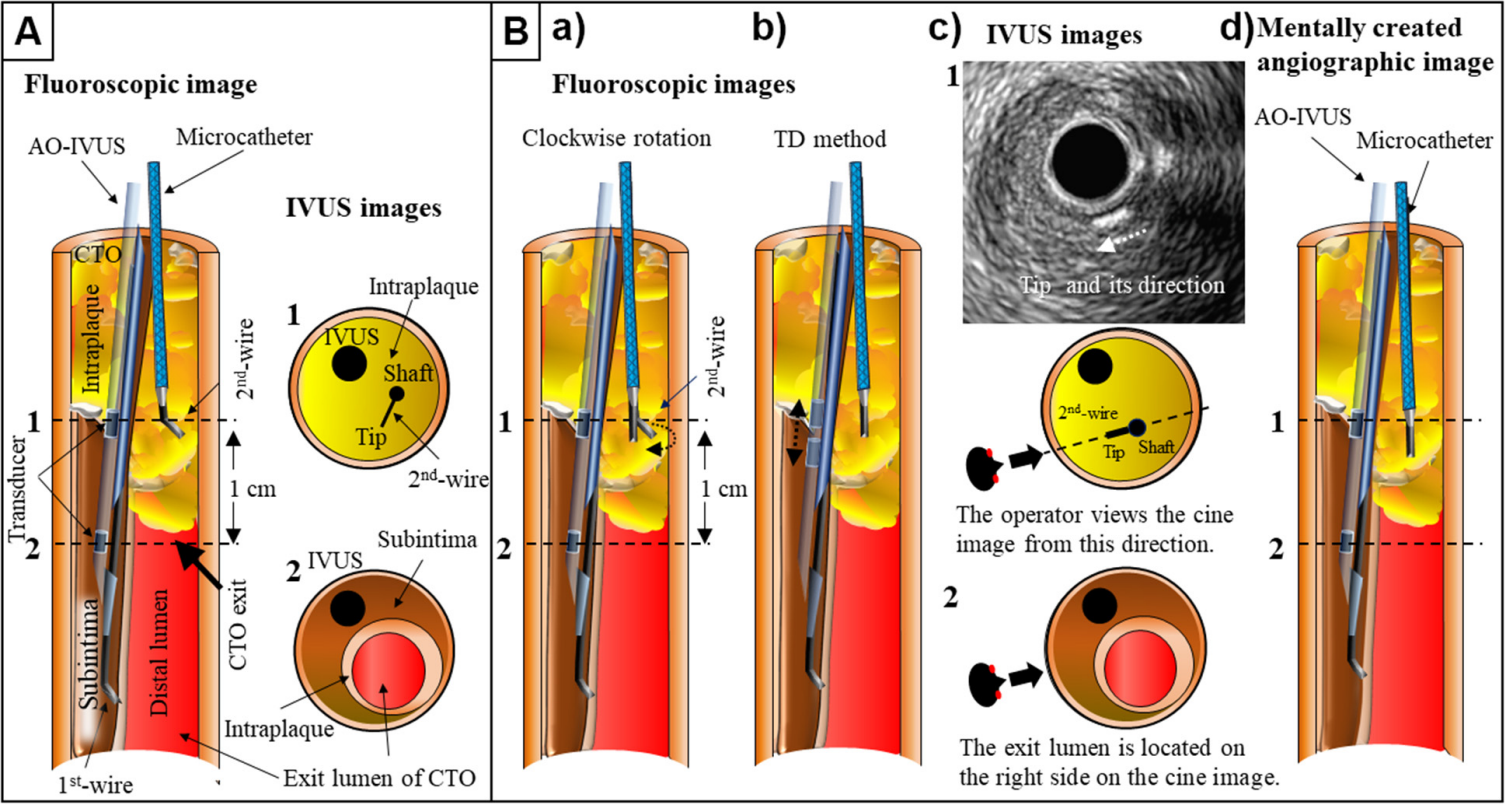
Transfer of Positional Information From IVUS Images to Fluoroscopic Images (A) Fluoroscopic and IVUS images of the second guidewire inside the CTO lesion 1 cm before the transitional site of the intimal and subintimal spaces. (B) Fluoroscopic images (a, b) and IVUS images (c) during the TD method, and (d) mentally created vascular image after the TD method. The numbers coincide with the numbered IVUS position images. Abbreviations as in Figure 1.

We used AO-IVUS in all cases of IVUS-guided wiring. First, we discuss how to recognize the location of the target (intraplaque, exit lumen of the CTO, etc), which can be visualized by IVUS, on the angiographic image. We also used the TD method, as shown in Figure 3. It is difficult to recognize the location of the target, such as the exit lumen of the CTO, which can be visualized by IVUS, on the angiographic images (Figure 3A). The second guidewire was advanced 1 cm before the transitional site of intraplaque area and subintimal space. On the angiographic image, the apex of the tip of the second guidewire faced the right (Figure 3B-a) and was rotated clockwise to place the apex of the tip directly facing the operator (Figures 3B-a and 3B-b). The TD method was then performed to determine the direction from which the operator was observing the angiographic image on the IVUS image (Figure 3B-b). On the IVUS image, the apex of the tip was directed toward the 8 o'clock position, and we were therefore observing the angiographic image from the 8 o'clock position on the IVUS image (Figure 3B-c-1). It was then simply recognized that the intraplaque area and exit lumen were located on the right side (Figure 3B-c-2, mentally created angiographic image).

First, we attempted to pass the guidewire through the intraplaque routes by the TD method (TD–intraplaque tracking), and if difficult, changed to TD-ADR to pass the guidewire through the re-entry route (Central Illustration B). During guidewire navigation, the tip and its direction were always visualized by moving the IVUS transducer back and forth at the guidewire tip part by an experienced second operator to visually construct the 3D image of the guidewire inside the vessel. In TD–intraplaque tracking, the guidewire tip was accurately guided to the target through the intraplaque route under direct visualization, whereas the transducer was always advanced in accordance with the advancement of the guidewire tip. In TD-ADR, the above-mentioned IVUS observation using TD method was also performed, and the wall between the subintima and the true lumen was punctured at the intended site in an exactly vertical direction. We attempted to create the re-entry just beyond the CTO exit. However, if puncture was difficult, we changed to puncture at the distal part. In September 2022, we found that TD-ADR at the CTO body was also possible.^15^ Since then, we have performed puncture at the CTO body when attempting to shorten the length of subintimal passage or when the CTO exit was located at the bifurcation site. Since the approval of CP12ST wire in August 2021, CP12ST wire has been used in all TD-ADR cases.

**Figure 4:**
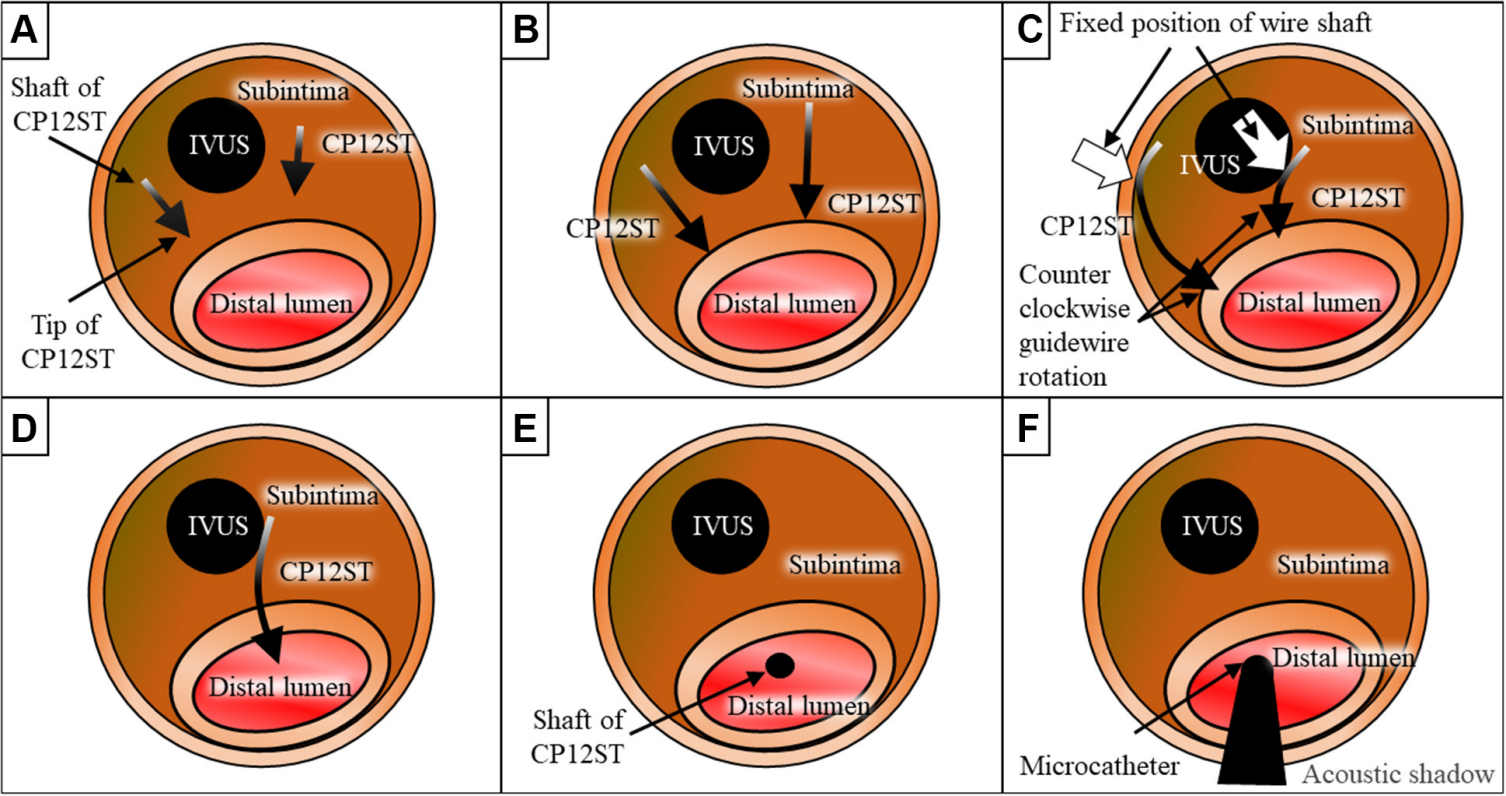
Creation of the Re-Entry Under IVUS Observation in TD-ADR (A to F) Illustrations of IVUS images during TD-ADR. CP12ST = Conquest Pro 12 Sharpened Tip; other abbreviations as in Figure 1.

Details of the TD-ADR procedure follow (Figure 4). Similar to Stingray-based ADR, TD-ADR uses the stick-and-swap technique, but based on IVUS guidance rather than angiographic guidance.

1. Formation of an adequate second curve (Figures 4A and 4B): The puncture guidewire is advanced through the microcatheter, and how the apex of the guidewire tip can hit the wall is checked by IVUS observation. From the IVUS information, an additional second curve can be adequately formed, if necessary, such that the apex of the guidewire tip can hit the wall vertically on the short-axis view of the IVUS image.
2. Fixing the guidewire shaft in the subintimal space (Figure 4C): Unlike TD–intraplaque tracking, it is difficult to fix the guidewire in the subintimal space. Therefore, in addition to forming an adequate second curve (step 1), it is necessary to consider the position and rotational direction of the guidewire for the shaft to be fixed against the IVUS catheter or vessel wall during puncture.
3. Stick (Figure 4D): The wall is punctured at the intended site in a vertical direction on the short-axis view of the IVUS image. In addition, a vertical puncture on the long-axis view can be recognized to some extent through fluoroscopy.
4. Swap (Figures 4E and 4F): Once the tip has penetrated the wall, it is advanced about 5 mm while keeping the apex of the tip inside the distal lumen. Then, the microcatheter is advanced into the lumen, and the tip of the microcatheter can be detected by the acoustic shadow under IVUS observation. The puncture guidewire is then changed to a soft guidewire. In addition, if it is difficult to pass the microcatheter after a successful puncture with the guidewire, consider using the side branch balloon anchor method or the GP-Lock method.^19^

### Study definition including IVUS analysis

CTO was defined as a totally occluded lesion with an estimated duration of at least 3 months with TIMI flow grade 0. The J-CTO (multicenter CTO registry of Japan) score was applied as reported previously.^20^ The angiographic classifications, such as collateral connection grade, CTO entry type, calcification, bending, and lesion length, were defined as reported previously.^21^ A definition of a retrograde approach was any attempt to advance a wire through a retrograde channel. Procedural success was defined as when both the guidewire and balloon crossed the occluded lesion completely, successfully dilating the occluded artery, and achieving restoration of antegrade flow (TIMI flow grade 3) with <50% residual stenosis on final angiography. Significant side branch occlusion beyond the CTO lesion was defined as occlusion of a side branch with a vessel diameter of 1.5 mm or more by angiography. In-hospital major adverse cardiac and cerebrovascular events consisted of cardiac and noncardiac death, Q-wave myocardial infarction, non–Q-wave myocardial infarction, target vessel failure followed by emergent target vessel revascularization with PCI or coronary artery bypass graft, and stroke. Myocardial infarction was defined as an increase in creatine kinase level to more than twice the upper limit of normal. Complications during the procedure consisted of vessel perforations recognized by the extravasation of contrast media by the guidewire without tamponade at the CTO site and vessel perforations leading to cardiac tamponade.

The procedural time for Stingray-based ADR was the time from delivering the Stingray balloon into the lesion until successful re-entry and advancement of the guidewire into the distal lumen. The procedural time for TD-ADR was the time from delivering the AO-IVUS into the lesion until successful re-entry and advancement of the guidewire into the distal lumen. The IVUS data were sent to Sakurabashi Watanabe Hospital and were analyzed by an independent experienced IVUS data analyst. The wall thickness at the successfully punctured site was measured by IVUS. When the CTO body was punctured, the farthest distance that the guidewire tip reached from the puncture site during puncture was defined as the wall thickness that could be punctured. The true lumen cross-sectional area was measured and the ratio of the true lumen area to the vessel area was calculated as the true lumen cross-sectional area divided by the external elastic membrane cross-sectional area at the successfully punctured site. The length of subintimal passage beyond the CTO exit was the distance from the CTO exit site to the re-entered distal lumen site measured under IVUS observation. However, in the case of re-entry from the CTO body in TD-ADR, the length of subintimal passage was defined as 0 mm.

### Statistical analysis

Continuous data were evaluated using the quantile-quantile plot and Kolmogorov-Smirnov test to check normality of continuous measures. Continuous data are expressed as the mean ± SD or median (Q1-Q3), whereas categorical values are expressed as percentages. Continuous data were compared using the unpaired Student’s *t*-test (for normally distributed data) or the Mann–Whitney *U* test (for non-normally distributed data). Frequency functions were assessed using Fisher exact test and the chi-square test. In all analyses, *P* < 0.05 was taken to indicate statistical significance. All statistical analyses were performed using EZR software (version 1.61, Saitama Medical Center, Jichi Medical University), which is a graphical user interface for R (R Foundation for Statistical Computing).

## Results

### Demographic and angiographic characteristics

Table 1 presents a summary of patient and lesion characteristics. There were no significant differences in the demographic or angiographic characteristics between the 2 groups.

**Table 1:** Demographic and Angiographic Characteristics

	Stingray-ADR by CP20 Wire Group (n = 27)	TD-ADR by CP12ST Wire Group (n = 27)	*P* Value
Demographic characteristics			
Age, y	71.1 ± 8.2	66.1 ± 12.7	0.095
Male	23 (85)	23 (85)	1.000
Clinical presentation			
Asymptomatic	13 (48)	14 (52)	0.785
Stable angina	14 (52)	13 (48)	
History of CABG	3 (11)	4 (15)	1.000
Coronary risk factor			
Hypertension	24 (89)	22 (81)	0.704
Diabetes mellitus	18 (67)	13 (48)	0.271
Dyslipidemia	23 (85)	22 (81)	1.000
Smoker	12 (44)	15 (56)	0.786
PAD	3 (11)	5 (19)	0.704
eGFR (<45 mL/min/1.73 m^2^)	3 (11)	4 (15)	1.000
Hemodialysis	2 (7)	2 (7)	1.000
LVEF <35%	3 (11)	8 (30)	0.175
Angiographic characteristics			
Target vessel			
RCA	8 (30)	8 (30)	0.806
LAD	9 (33)	7 (26)	
LCX	10 (37)	12 (44)	
LMT	0 (0)	0 (0)	
CABG	0 (0)	0 (0)	-
Collateral Werner's score			
CC0	6 (22)	4 (15)	0.486
CC1	14 (52)	12 (44)	
CC2	7 (26)	11 (41)	
CTO entry types			
Blunt	19 (70)	22 (81)	0.526
Severe calcification	16 (60)	18 (67)	0.779
Bending (>45°)	7 (26)	11 (41)	0.387
Occluded length (>20 mm)	13 (48)	19 (70)	0.166
Reattempted lesion	8 (30)	7 (26)	1.000
ISR	1 (4)	0 (0)	1.000
J-CTO score	2.0 (2.0-3.0)	3.0 (2.0-3.0)	0.062

Values are mean ± SD, n (%), or median (Q1-Q3).

ADR = antegrade dissection re-entry; CABG = coronary artery bypass graft; CC = collateral connection grade; CP12ST = Conquest Pro 12 Sharpened Tip; CP20 = Conquest 8–20; CTO = chronic total occlusion; eGFR = estimated glomerular filtration rate; ISR = in-stent restenosis; J-CTO score = Japanese chronic total occlusion score; LAD = left anterior descending coronary artery; LCX = left circumflex coronary artery; LMT = left main trunk; LVEF = left ventricular ejection fraction; PAD = peripheral arterial disease; RCA = right coronary artery; TD-ADR = tip detection–antegrade dissection re-entry.

### Comparison of procedural outcomes between Stingray-ADR and TD-ADR

**Figure 5:**
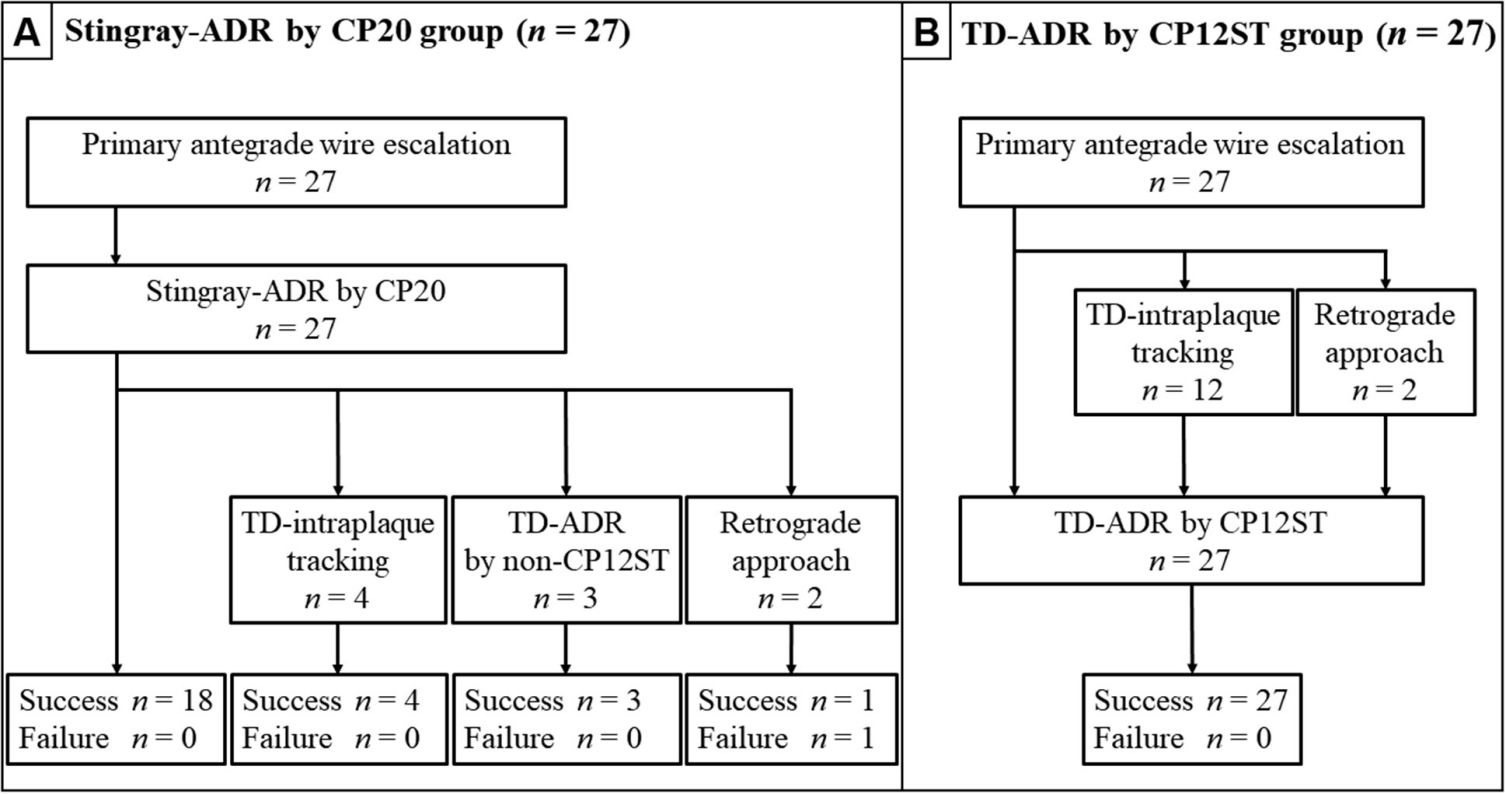
Flow Diagram of Stingray-ADR by CP20 Wire and TD-ADR by CP12ST Wire Flow diagrams of the procedures (A) in the Stingray-ADR by Conquest 8-20 (CP20) wire group and (B) in the TD-ADR by CP12ST wire group. (A) Stingray-based ADR was performed in all the 27 cases just after 13 the primary antegrade wire escalation. Stingray-based ADR was successful in 18 cases; however, 9 cases required other methods, and only 1 case was unsuccessful. (B) TD-ADR was performed in 13 cases just after the primary antegrade wire escalation. However, TD-ADR was performed after TD–intraplaque tracking or the retrograde approach in 14 cases, and TD-ADR was successful in all cases. Abbreviations as in Figures 1 and 4.

Figure 5 shows flow diagrams of the procedures in the 2 groups. In the Stingray-ADR by CP20 wire group, Stingray-based ADR was performed in all the 27 cases just after the primary antegrade wire escalation. Stingray-based ADR was successful in 18 cases; however, 9 cases required other methods, mainly TD methods, and only 1 case was unsuccessful. In the TD-ADR by CP12ST wire group, TD-ADR was performed in 13 cases just after the primary antegrade wire escalation. However, TD-ADR was performed after TD–intraplaque tracking in 12 cases, and TD-ADR was performed after the retrograde approach in 2 cases, and TD-ADR was successful in all cases.

Table 2 presents the procedural outcomes. The total procedure time was significantly shorter in the TD-ADR by CP12ST wire group than in the Stingray-ADR by CP20 wire group (median: 145.0 [Q1-Q3: 118.0-240.0] minutes vs 185.0 [Q1-Q3: 159.5-248.0] minutes, respectively; *P* = 0.028). Radiation absorbed dose and amount of contrast medium were also significantly reduced in the TD-ADR by CP12ST wire group compared with the Stingray-ADR by CP20 wire group. ADR-related procedural data were significantly improved in the TD-ADR by CP12ST wire group compared with the Stingray-ADR by CP20 wire group (success rate of the ADR procedure: 100% [n = 27 of 27] vs 67% [n = 18 of 27], respectively; *P* = 0.002; procedural time for ADR: median: 11.0 [Q1-Q3: 6.0-17.5] minutes vs 31.0 [Q1-Q3: 21.8-39.5] minutes, respectively; *P* < 0.001; number of punctures for re-entry: median: 2.0 [Q1-Q3: 1.0-3.0] vs 4.0 [Q1-Q3: 2.0-7.0], respectively; *P* = 0.008; and length of subintimal passage: median: 4.0 [Q1-Q3: 2.0-6.5] mm vs 14.0 [Q!-Q3: 8.5-16.0] mm, respectively; *P* < 0.001.). In addition, thicker walls could be punctured in the TD-ADR by CP12ST wire group compared with the Stingray-ADR by CP20 wire group (median: 0.5 [Q1-Q3: 0.4-0.7] mm vs 0.3 [Q1-Q3: 0.2-0.4] mm, respectively; *P* < 0.001) and there was 1 case in which the CTO body was successfully punctured in the TD-ADR by CP12ST wire group. There were no significant differences in the in-hospital major adverse cardiac and cerebrovascular events or complication rates between the 2 groups (Table 3).

**Table 2:** Procedural Characteristics and Outcomes

	Stingray-ADR by CP20 Wire Group	TD-ADR by CP12ST Wire Group	*P* Value
Total cases of ADR	27	27	
Frequency of use of 8-F guide catheter for antegrade approach	26 (96)	27 (100)	1.000
Frequency of IVUS-guided TD–intraplaque tracking before ADR procedures		12 (44)	
Predilatation before ADR procedures			
Corsair's bougie only	20 (74)	13 (48)	0.093
Addition of balloon dilatation with small-diameter balloon	6 (22)	13 (48)	0.083
Rotablation	1 (4)	1 (4)	1.000
Microcatheter in TD-ADR			
Corsair		8 (30)	
Finecross		19 (70)	
Procedural time for ADR, min	29.0 (23.0-41.0)	11.0 (6.5-17.5)	<0.001
Total procedure time, min	185.0 (159.5-248.0)	145.0 (118.0-204.0)	0.028
RAD, mGy	3885.0 (2448.5-5392.5)	3056.0 (2153.0-3726.5)	0.038
Contrast dose, mL	200.0 (135.0-240.0)	130.0 (97.5-192.5)	0.011
Stent length, mm	51.5 (40.5-59.8)	38.0 (27.0-50.0)	0.010
Success rate of ADR procedure	18 (67)	27 (100)	0.002
Success rate throughout the procedure	26 (96)	27 (100)	1.000
Successful cases of ADR	18	27	
Procedural time for ADR, min	31.0 (21.8-39.5)	11.0 (6.5-17.5)	<0.001
No. of punctures for re-entry	4.0 (2.0-7.0)	2.0 (1.0-3.0)	0.008
Wall thickness at successful punctured site, mm	0.3 (0.2-0.4)	0.5 (0.4-0.7)	<0.001
True lumen cross-sectional area at the punctured site, mm^2^		2.5 (1.9-3.3)	
Ratio of the true lumen area to the vessel area at the punctured site		0.3 (0.3-0.4)	
Side branch occlusion	3 (17)	0 (0)	0.058
Length of subintimal passage, mm	14.0 (8.5-16.0)	4.0 (2.0-6.5)	<0.001

Values are n (%) or median (Q1-Q3).

IVUS = intravascular ultrasound; RAD = radiation absorbed dose; other abbreviations as in Table 1.

**Table 3:** In-Hospital MACCE and Complications During the Procedure

	Stingray-ADR by CP20 Wire Group (n = 27)	TD-ADR by CP12ST Wire Group (n = 27)	*P* Value
MACCE	0 (0)	0 (0)	-
Cardiac death	0 (0)	0 (0)	-
Noncardiac death	0 (0)	0 (0)	-
QMI	0 (0)	0 (0)	-
Non-QMI	1 (4)	0 (0)	1.000
Emergent target vessel revascularization with PCI or CABG	0 (0)	0 (0)	-
Stroke	0 (0)	0 (0)	-
Complications during the procedure			
Vessel perforation by the guidewire	0 (0)	0 (0)	-
Cardiac tamponade	0 (0)	0 (0)	-

Values are n (%).

MACCE = major adverse cardiac and cerebrovascular event(s); PCI = percutaneous coronary intervention; QMI = Q-wave myocardial infarction; other abbreviations as in Table 1.

### Representative cases of TD-ADT by CP12ST wire

**Figure 6:**
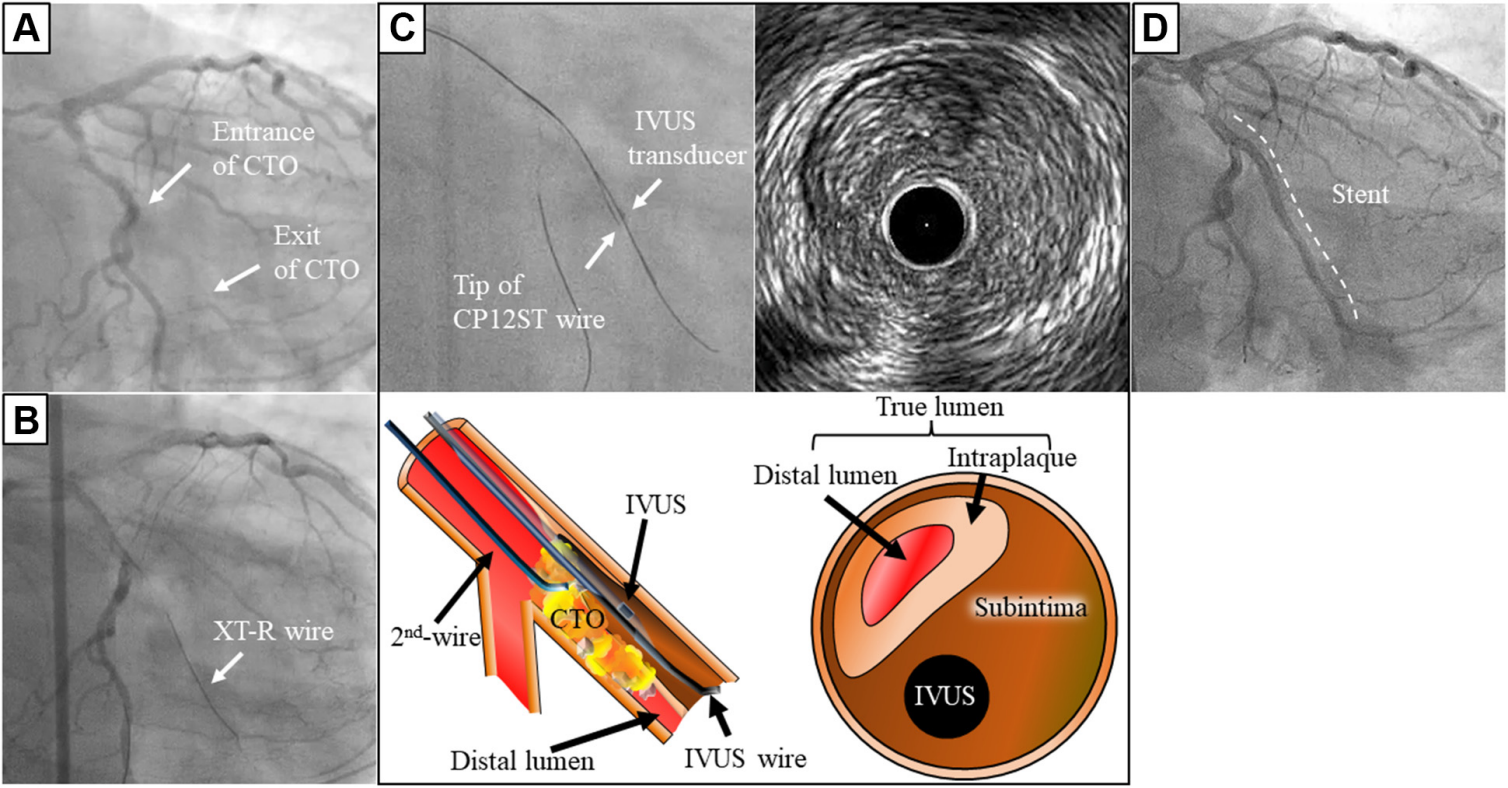
Angiographic and IVUS Images During the Procedures Angiographic images (A) prior to the procedure and (B) during antegrade wire escalation, (C) angiographic and IVUS images and corresponding illustrations during TD-ADR, and (D) angiographic image after the procedure. Abbreviations as in Figures 1 and 4.

**Figure 7:**
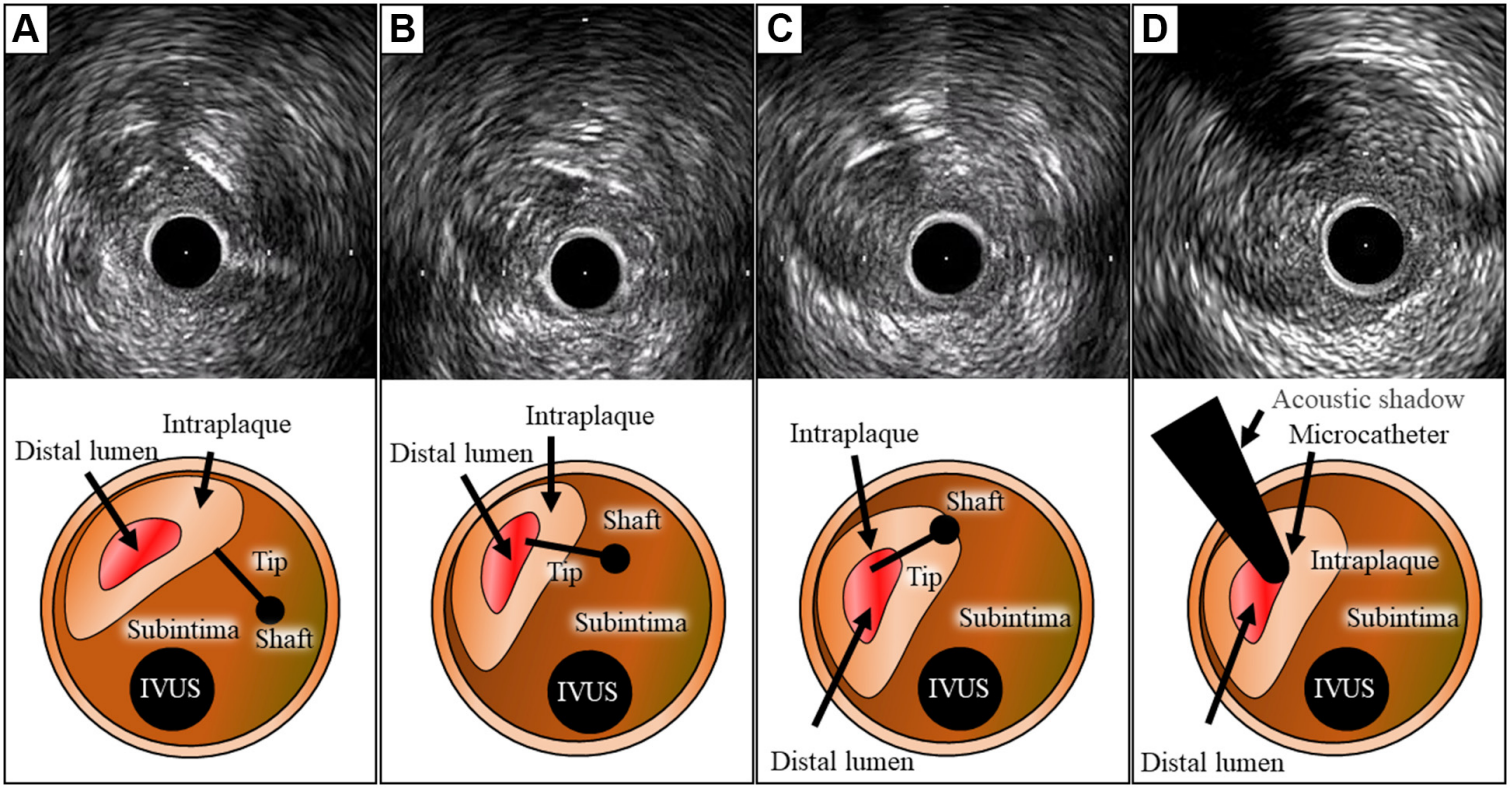
IVUS Images During TD-ADR and Corresponding Illustrations (A) The tip of the guidewire was vertically touching the wall of the true lumen. (B) One-half of the length of the tip enters the wall. (C) The tip and shaft entered the true lumen. (D) The microcatheter entered the distal lumen in the true lumen (Video 1). IVUS images during TD-ADR. Abbreviations as in Figure 1.

A 54-year-old man with effort angina pectoris due to a CTO lesion in the left circumflex coronary artery was treated using TD-ADR by CP12ST wire (Figure 6A). After insertion of an 8-F guide, an XT-R wire could be advanced into the CTO lesion (Figure 6B). However, AO-IVUS observation revealed that it entered the subintima 1 cm beyond the CTO entrance due to severe calcification (Figure 7A), whereas there was a true lumen without calcification 5 mm beyond the transition site. The true lumen could not be visualized angiographically, so we selected AO-IVUS–based TD-ADR by CP12ST wire instead of Stingray-based ADR. The CP12ST wire supported by a Corsair microcatheter was advanced to the re-entry site where we attempted re-entry (Figure 6C). The TD method allowed the tip of the CP12ST wire to puncture the wall of the true lumen in an exactly vertical direction, resulting in successful re-entry even though the true lumen had collapsed and had thick plaque (Figures 7C and 7D). After advancing the Corsair microcatheter into the true lumen, CP12ST wire was changed to a soft guidewire, which was advanced into the distal part (Figure 7D, Video 1). Normal antegrade blood flow was achieved after stent implantation (Figure 6D).

**Figure 8:**
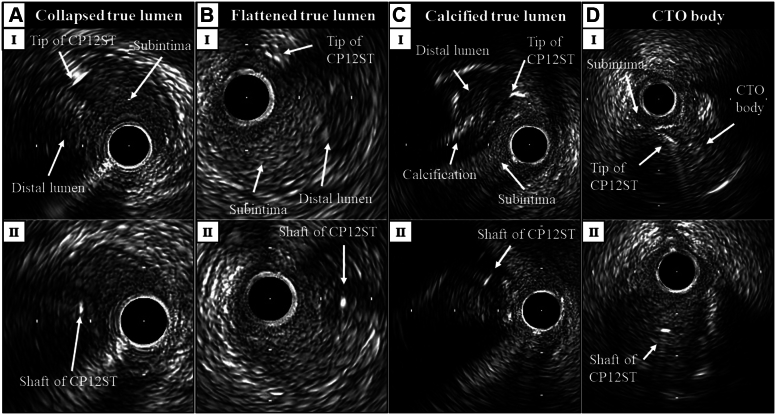
IVUS Images During TD-ADR for 4 Other Impressive Cases IVUS images from the start of vertical puncture (I) to successful re-entry puncture (II) during TD-ADR for (A) the case of the collapsed true lumen (Video 2), (B) the case of the flattened true lumen (Video 3), (C) the case of the true lumen with calcifications in the wall (Video 4), and (D) the previously reported case of the CTO body (Video 5).^15^ Abbreviations as in Figures 1 and 4.

Figure 8 (Video 2, Video 3, Video 4, Video 5) shows 4 other impressive TD-ADR puncture images.

## Discussion

TD-ADR allowed reliable re-entry in CTO-PCI by puncturing the wall between the subintima and the true lumen at the intended site in a vertical direction under IVUS observation. Therefore, TD-ADR by CP12ST wire significantly shortened the length of the subintimal passages, reduced the total procedural time, and increased the success rate of ADR compared with Stingray-ADR by CP20 wire.

### Why is TD-ADR by CP12ST wire a useful re-entry method over Stingray-ADR by CP20 wire?

In general, the current device-based ADR is not recommended in situations where the distal lumen cannot be visualized by contrast medium, the CTO exit is located at a bifurcation lesion, the plaque at the puncture site is thick, and the true lumen is collapsed or small.^2^^,^^18^ However, TD-ADR by CP12ST wire overcomes all of these situations because IVUS allows visualization of all the true lumen, re-entry from the CTO body is possible, the CP12ST wire has high penetration force, the TD method allows puncture regardless of the target size, and the Seldinger method^22^ under IVUS observation makes it possible to accurately insert the tip of the microcatheter into the true lumen. That is, combining TD-ADR with CP12ST wire makes re-entry possible from anywhere regardless of the wall thickness except at sites of calcification. The disadvantage of TD-ADR is that the current device-based ADR can be performed by 1 operator, but TD-ADR requires an assistant who can perform appropriate IVUS operations as a second operator along with the rest of the team.

The main operators were A.O. and Y.R., who are the Japanese CTO-PCI expert registry operators with more than 20 years of CTO-PCI experience, and K.T., S.S., and M.I. who had more than 5 years of CTO-PCI experience. For both Stingray-based ADR and TD-ADR, 1 of these 5 operators was the first or second operator. Fifteen of 19 TD-ADR cases at Sakurabashi Watanabe Hospital were treated by A.O. and K.T. in pairs. As mentioned in the Methods, there are several techniques specific to TD-ADR. However, TD-ADR does not have the black box factor in guidewire manipulation, and an operator simply advances the guidewire toward the target under visual navigation. Therefore TD-ADR can be performed by an operator with approximately 5 years of CTO-PCI experience.

Before the approval of the CP12ST wire, 4 cases were treated by TD-ADR by non-CP12ST wire. The non-CP12ST wires used were GAIA Next 3 wire (Asahi Intecc Co, Ltd)^12^ in 1 case and CP20 wire^13^ in 3 cases. In all cases, TD-ADR was successful, and the median procedural time for ADR was 11.0 (Q1-Q3: 6.0-17.5) minutes. Mechanically, TD-ADR is better with the wire that has more penetrating force, therefore, following approval of CP12ST wire, we shifted to using CP12ST wire. When the CP12ST wire was approved in August 2021, Stingray-based ADR had already been changed to TD-ADR at Sakurabashi Watanabe Hospital and the 3 other facilities. Therefore, the CP12ST wire was not used in Stingray-based ADR in the present study, and therefore the effects of Stingray-ADR by CP12ST wire are not known. As the TD-ADR method is a highly accurate procedure compared with Stingray-based ADR, only TD-ADR by CP12ST wire has been performed in all ADR procedures in our daily practice. In the TD-ADR groups, an 8-F guide catheter was used in all cases. AO-IVUS can be inserted into a 7-F guide catheter with a microcatheter including a Corsair for the puncture guidewire, and therefore a 7-F guide catheter can also be used for the TD-ADR procedure.

### TD-ADR by CP12ST wire will affect the CTO-PCI algorithms

In the 2 periods of the present study, the ADR rate clearly increased, with a TD-ADR rate of 27 of 187 (14.4%) and a Stingray-based ADR rate of 27 of 317 (8.5%). This suggests that a paradigm shift has occurred in the strategy of CTO-PCI. As shown in the present study, the IVUS-guided TD method, TD–intraplaque tracking^7^, ^8^, ^9^, ^10^ and TD-ADR,^12^^,^^13^^,^^15^ is a highly accurate guidewire navigation technique, and we hope that the CTO-PCI algorithms will be changed in the future.^11^ In antegrade wire escalation, when the guidewires cannot pass through the CTO exit due to advancement into the subintimal space, or intraplaque area other than the distal lumen, the retrograde approach will be selected if there are promising interventional retrograde channels. However, in more situations than before, AO-IVUS will be inserted into the CTO lesions, followed by TD–intraplaque tracking and TD-ADR. The retrograde approach is still necessary when the guidewires cannot be advanced into the CTO lesion or, although we have never experienced it, when the lesions are completely calcified and puncture sites cannot be found by IVUS observation.

In order to perform the TD method including TD-ADR, a short-tip pull back IVUS such as AO-IVUS is necessary, although additional medical expenses are required. We hope that AO-IVUS and CP12ST wire will also become available outside of Japan and the TD method will be widely adopted around the world.

### Study limitations

TD-ADR requires several techniques specific to this procedure and an assistant who can perform appropriate IVUS operations as a second operator. Stingray-based ADR was performed by CP12 wire but TD-ADR was performed by CP12ST wire. Therefore, a comparative study of Stingray-ADR by CP12ST wire and TD-ADR by CP12ST wire is necessary to determine whether TD-ADR is effective against Stingray-based ADR. The present study was based on retrospective data analysis of a relatively small number of patients (Comparison between number of cases <50). Further, multicenter and prospective randomized studies are needed to evaluate whether the TD-ADR by CP12ST wire method is effective and feasible for use in daily clinical practice.

## Conclusions

TD-ADR by CP12ST might be an innovative method to standardize highly accurate ADR in CTO-PCI.

Perspectives **COMPETENCY IN MEDICAL KNOWLEDGE:** We devised the TD method and developed AO-IVUS to standardize IVUS-based 3D wiring for intraplaque tracking in CTO-PCI. Recently, we found that the TD method also allows ADR (TD-ADR) by puncturing the wall at the intended site in an exactly vertical direction and reported several cases. We compared the procedural results of Stingray-ADR by CP20 wire and TD-ADR in CTO-PCI by CP12ST wire and showed the usefulness of TD-ADR by CP12ST wire in comparison to Stingray-ADR by CP20 wire.

**TRANSLATIONAL OUTLOOK:** TD-ADR will standardize highly accurate ADR in CTO-PCI in Japan. Further multicenter and prospective randomized studies are required to determine the effectiveness of TD-ADR worldwide. A short-tip pull back IVUS such as AO-IVUS is not available outside Japan, and we hope that this device lag issue will be resolved soon.

## Funding Support and Author Disclosures

Dr Okamura has received speaker fees from Terumo Corp. Dr Tsuchikane has received consulting fees from Asahi Intecc, Boston Scientific, and Kaneka. All other authors have reported that they have no relationships relevant to the contents of this paper to disclose.

## Appendix

Video 1 TD-ADR for the True Lumen: IVUS Video Image From the Start of the Vertical Puncture, Successful Re-entry Puncture, and the Insertion of the Microcatheter

Video 2 TD-ADR for the Collapsed True Lumen: IVUS Video Image From the Start of the Vertical Puncture, Successful Re-entry Puncture, and the Insertion of the Microcatheter

Video 3 TD-ADR for the Flattened True Lumen: IVUS Video Image From the Start of the Vertical Puncture, Successful Re-entry Puncture, and the Insertion of the Microcatheter

Video 4 TD-ADR for the True Lumen With Calcifications in the Wall: IVUS Video Image From the Start of the Vertical Puncture to Successful Re-entry Puncture

Video 5 TD-ADR for the CTO Body: IVUS Video Image From the Start of the Vertical Puncture, Successful Re-entry Puncture, and the Insertion of the Microcatheter
